# Editorial: Unsafe human behavior at construction sites

**DOI:** 10.3389/fpsyg.2022.1023957

**Published:** 2022-10-20

**Authors:** Qingfeng Meng

**Affiliations:** School of Management, Jiangsu University, Zhenjiang, China

**Keywords:** summary, topic, unsafe behavior, construction site, worker

## Overview of research contents

Based on the analysis of the papers collected by the special journal, it is found that most scholars like to explore the complex influencing factors of unsafe behaviors on the construction site and their action paths to unsafe behaviors. For example, Ni et al. used grounded theory and system dynamics to explore the formation mechanism and dynamic evolution of unsafe behaviors of the new generation of construction workers in China's construction industry. Qie and Yan discussed the complex influencing factors and action paths of unsafe accidents in subway construction sites. Li P et al. used a latent Dirichlet (LDA) assignment model to identify the unsafe behaviors of construction workers and their influencing factors, and then, with the help of social network analysis, determined the importance of the influencing factors and their relationships. Zhou et al. analyze the motives of core enterprises to participate in safety management in supply chain from a behavioral perspective by using in-depth interviews and grounded theory, that is, the internal and external influencing factors of core enterprises' work safety management behaviors in supply chain and their mechanisms on enterprise management behavior decisions are explored in depth from a microscopic perspective.

In addition, some scholars also like to explore the mechanism of unsafe behaviors of workers from certain specific angles. For example, Li W et al. constructed a safety production behavior mechanism model based on the theory of planned behavior (TPB). Zhao et al. explored the impact of safety leadership on employee safety engagement. Ye et al. used self-exhaustion and self-efficacy as mediating variables to explore the relationship between safety stressors and construction workers' safety performance. Ji et al. considered multiple heterogeneities and studied the competition incentive mechanism of construction workers' safety behavior. Li Z et al. established an Agent model under the awareness of formal rules and herd mentality, and studied the unsafe behavior of construction workers. Ning et al. used an evolutionary game model to describe the decision-making interactions between the government and construction enterprises under the enterprise entity responsibility and third-party participation mechanisms.

Finally, some scholars have also made corresponding research on the prevention and control of workers' unsafe behaviors. For example, Peng and Zhang used an evolutionary game approach to provide managers with advice on how to handle the situation following a conflict among construction workers. Zhu et al. took the incentive and punishment mechanism into consideration and established an evolutionary game model to improve the effectiveness of construction safety management. Zijian et al. extracted recurring unsafe behavior scenarios in high-speed fall accidents through Bayesian network derivation based on a structurally constrained PC algorithm. Cong et al. developed a theoretical model for evaluating the communication performance of construction workers to enhance informal safety communication in the workplace.

To sum up, it can be seen that the research on unsafe behavior of workers shows a trend of diversification. Whether it is research content or research method, the research content mainly involves the formation mechanism and prevention strategies of the influencing factors of unsafe behavior, and the research methods mainly include Structural Equation Model, Evolutionary Game, Bayesian Network and Agent-Based Modeling.

This special issue is expected to provide valuable insights for building stakeholders to improve safety performance in practice, and at the same time, it guides future research in this research area.

## Author contributions

The author confirms being the sole contributor of this work and has approved it for publication.

## Conflict of interest

The authors declare that the research was conducted in the absence of any commercial or financial relationships that could be construed as a potential conflict of interest.

## Publisher's note

All claims expressed in this article are solely those of the authors and do not necessarily represent those of their affiliated organizations, or those of the publisher, the editors and the reviewers. Any product that may be evaluated in this article, or claim that may be made by its manufacturer, is not guaranteed or endorsed by the publisher.

